# Food safety knowledge, attitudes, and eating behavior in the advent of the global coronavirus pandemic

**DOI:** 10.1371/journal.pone.0261832

**Published:** 2021-12-31

**Authors:** Zhe Liu, Anthony N. Mutukumira, Cong Shen

**Affiliations:** 1 School of Management, Henan University of Technology, Zhengzhou, Henan, P. R. China; 2 School of Food and Advanced Technology, Massey University, Auckland, New Zealand; Univerza v Mariboru, SLOVENIA

## Abstract

The objective of this study was to evaluate the relationships of food safety knowledge, attitude and eating behavior of consumers during national lockdowns in the advent of the COVID-19 pandemic. A total of 157 respondents completed the online survey using a structured questionnaire worldwide. Overall, the respondents exhibited good attitude and good knowledge towards public health including food safety especially on the importance of social distancing, mask wearing, well-balanced diet, physical exercise and personal hygiene, such as hand washing during the pandemic lockdowns. A Structural Equation Modeling (SEM) was used to test the relationships among food safety knowledge, attitude and behavior under the pandemic conditions. Results showed that attitude towards food safety under the coronavirus pandemic and lockdowns positively affected the eating behavior of the respondents, which exhibited a high β (0.686) among the variables tested (p<0.05). Food safety knowledge was apparently not affected by the food safety behavior of the respondents.

## 1. Introduction

The COVID-19 pandemic that emerged in 2019 has imposed huge consequences on public health and economic losses, which continue to affect many aspects of our daily life globally. According to World Health Organization (WHO), there have been over 227 million confirmed cases of COVID-19, including nearly 5 million deaths as of September 2021 [1]. Possible reasons for the crisis included the continued spread of transmission of variants of concern (VOCs), relaxation of public health and social measures (PHSM) and human fatigue around adhering to PSHM measures [2]. That means basic public health measures, such as Nucleic Acid Amplification Tests, contact tracing, isolation, supervised quarantine, optimal care, avoiding crowds, exercising, physical distancing, routine hand hygiene, wearing masks, and ensuring efficient ventilation of dwelling, remain very important under current pandemic.

One year has elapsed since the virus was first reported and its characteristics are not still well understood. The source of the SARS-CoV-2 is still unknown, transmission channels and incubation time continue to cause challenges to national authorities and international organizations [3]. The early cases of COVID-19 were presumed to be linked to the Huanan Marine Market in Wuhan, and the possibilities of transmission to humans may not be excluded based on available data [4–6]. Further, the successful isolation of SARS-CoV-2 virus using trace back analysis from the seafood packaging surfaces indicated that the imported virus re-infected humans and may have caused the outbreaks through cold-chain transportation is all possible [7]. Food safety has been a topical issue during COVID-19 [8], with several studies conducted on challenges such as preventative measures, perception of risk, trust of food safety information, and consumer willingness to pay (WTP) [9–11]. Also, several assessments of knowledge, attitude, and practices of residents and healthcare workers have been reported from China, Malaysia, Pakistan, and Uganda during the COVID-19 pandemic [12–16]. Earlier studies have mainly reported on knowledge, attitude and behavior of consumers during the pandemic. Based on the published information, it is apparent that there is a gap on the relationships or interactions of the factors studied, which is the focus of the current study. Since the outbreak of COVID-19 pandemic consumers, national authorities and other organizations have been faced by a multitude of challenges including maintaining high standards of foods safety management practices. The challenges of food safety have been compounded by extended restricted movements (lockdowns), hence there is need to examine their inter-relationships during the pandemic. Thus, identifying the impact of the COVID-19 pandemic on consumers’ food safety knowledge, attitude and eating behavior under lockdown or restricted movements may be necessary to support effective inactivation strategies and generate data for future planning to effectively combat similar pandemics. The objective of the present study was to explore the relationships among food safety knowledge, attitude and eating behavior under the coronavirus pandemic and related lockdowns. In this study, food safety encompasses activities and actions that are associated with the supply or provision of food to the consumers (FAO/WHO, 1992) [17]. Therefore, the ‘relationships’ presented in this paper are interlinked to the access of safe food by consumers. Based on published reports, the present study is the first of its kind to present information on the three relationships mentioned here under the coronavirus pandemic.

## 2. Materials and methods

### 2.1 Model and sampling plan

**Fig 1:**
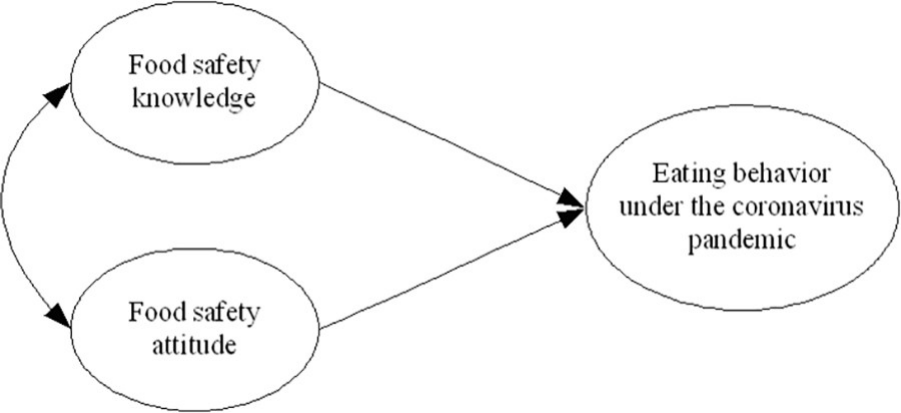
Model of relationships among food safety knowledge, attitude and eating behavior under the global coronavirus pandemic and varying lockdowns.

According to McIntosh et al., knowledge is associated with current practices, which in turn affects willingness to change current practices if it is acknowledged that current practices are unsafe [18]. Based on the cognitive-affective-behavior theory in social psychology, several studies have been published on the relationships among knowledge, attitude and behavior after Schwartz [19–22]. However, there seems to be a disparity between food safety knowledge and self-reported practices [23]. The reasons for cooking preferences may be unaffected by either knowledge or mass media exposure. It is interesting to note that food safety knowledge did not have a significant influence on food safety behavior [24]. In contradiction, other studies found that there was a small and positive effect or a significant and negative relationship between food safety knowledge and behavior [19, 25]. Thus, the relationship between knowledge and behavior can be considered as the extent to which the consumers are concerned about different eating behaviors regarding food safety issues. Attitude is an important psychological construct, which can influence and predict several behaviors [26]. There are six dominant factors of food safety consumer attitudes including chemical issues, health issues, spoilage issues, regulatory issues, deceptive practices, and assessment of ideal situations [27]. The relationship between attitude and behavior can be defined as the extent to which the consumers have the willingness to change behavior determined by perceptions and beliefs. Perceptions and beliefs are shaped by knowledge, which in turn is a product of exposure to information sources and personal effort in obtaining information [28]. Food safety attitude of the community was found to strongly affect the food safety behavior positively [24]. However, different attitudes of individuals do not necessarily lead to behaviors that increase the safety of the food consumed. In the present study, alternative hypotheses were formulated and are illustrated in Fig 1 based on the relationships suggested by Schwartz [19]:

H1: Food safety knowledge directly affects the healthy eating behavior under the coronavirus pandemic.
H2: Food safety attitude directly affects the healthy eating behavior under the coronavirus pandemic.
H3: There is a statistically significant relationship between food safety knowledge and attitude under the coronavirus pandemic.

As individuals that constitute the population of the study all over the world were not easily accessible, probability sampling was preferred [29]. In this study, a simple random sampling method was used to represent the population. Similar methods of conducting online surveys have been reported [30]. An online survey of food safety was conducted among 157 people under the coronavirus pandemic, from May 2020 to December 2020. During this period, various regions were experiencing different levels of lockdowns (restricted movements). The data of the research were collected through an online survey platform with a questionnaire.

### 2.2 Description of the design of the questionnaire

This study was conducted online during the global coronavirus (COVID-19) pandemic. The respondents were randomly selected through emails, social media platforms, personal contacts, as well as websites of professional bodies. The data of the research were collected through the professional online questionnaire platform of www.wjx.cn (S1 File), and the response rate was 100%. Questionnaire was developed and divided into 4 parts consisting of socio-demographic characteristics of the respondents, food safety knowledge, and attitude and eating behavior. Part I consisted of the demographic section, used to collect data on gender, age, composition of household, employment status, level of education, and location of the participants. Part II consisted of a scale on food safety knowledge, including 9 questions that tested the knowledge of the respondents of COVID-19 (5 items), and food safety (4 items). Parts III and IV evaluated the food safety attitudes using 14 questions on health issues under coronavirus pandemic, and 10 questions on changes in eating behavior of the participants, respectively. Each question consisted of 2 optional answers (“yes”, or “no”), which were intended to prevent the participants to select the correct answer by guessing. Each correct response or healthy habit was allocated 1 point and incorrect response 0 point.

### 2.3 Ethical statement

Collection of data using the online survey was covered by a Low-Risk Ethics Application (S2 File) granted by Massey University Ethics Committee, New Zealand. The survey was completely anonymous and voluntary as it did not request for personal details of respondents such as names, date of birth, region/country of residence, residential address, etc. Therefore, the survey did not have any potential ethical issues. In this regard, participants were not required to complete confidentiality or consent forms. In the section on survey instructions, participants were informed in writing that all data on their background (e.g. level education) and eating habits were anonymously collected and solely used for the purpose of the study. No minor was included in the survey.

### 2.4 Pilot study

The questionnaire was pilot tested and modified to be compatible with the online survey through websites, cellphone, email contacts and other remote contact platforms [29]. The online survey questionnaire was prepared through several stages. In the first stage, a draft questionnaire was prepared and moderated by several scientists who had knowledge and interest in the research area. In the second stage, the revised questionnaire was tested online with 25 university volunteer students followed by another round of revision. The third revised questionnaire was also tested followed by a final revised version which was used to collect the research data. The individuals involved in the development of the questionnaire were not included in the collection of substantive data used in the study. In a broader context, food safety, knowledge, attitude, and eating behavior are key components of modern food safety management systems as they contribute to food safety. Modern food safety incorporates these aspects under the aspirations of the 1992 Nutrition Convention in Rome (FAO/ WHO, 1992) [17]. The questions feature pertinent factors that are encountered in a social context in food safety management systems and practices.

### 2.5 Data collection

Data were collected using an online survey platform of www.wjx.cn (S1 File). The online questionnaire was available in two versions, in English and Chinese. The call for participation was made on social media using various platforms, email messages and professional networks available to the researchers. Online sample data (n = 157) were received from 14 countries and the data were considered sufficient for conducting a structural equation model (SEM) [31–34]. Data were retrieved from the completed online questionnaires and collated for analysis using various statistical tools.

A sampling strategy is considered adequate since it is not always possible to collect data from every unit of the population. Thus, determining an appropriate sample size is vital to draw valid conclusions from research findings [35]. However, there is no simple rule of thumb about sample size that works across all studies [36]. Recent developments suggest that researchers should determine sample size through power analysis which includes part of the largest number of predictors, effect size, and significance level [37–41]. G*Power is one of the various statistical programs used to perform power analysis and is often the first choice for business and social science studies [42, 43]. According to the most common recommended setting for social and business science studies [38], specifying the effect size at 0.15, α at 0.05, and power at 0.80 in the input parameters, the calculated G*Power estimated that the minimum sample size required for the hypothesized model was 68, as shown in the Supporting Information. Further, specifying power at 0.95, G*Power estimated that the minimum sample size required for the simple model was 107. In this study, data were collected over a longer period, thus they should be considered as cross-section, and representative data since the prevalence of coronavirus pandemic was global. In this primary study, there were only 3 latent variables in the hypothesized model, as shown in Fig 1. An extended version of the model that integrated moderators (i.e., countries, languages, Employment status) among food safety knowledge, attitudes, and eating behavior is recommended in further studies.

### 2.6 Statistical analysis

Data were analyzed by IBMSPSS (The Statistical Package for Social Sciences) for descriptive analysis, normality test, reliability analysis, Pearson’s correlation coefficient analysis and Exploratory Factor Analysis (EFA). Confirmatory Factor Analysis (CFA) and structural equation modeling were conducted by MPLUS 8.3 with confidence level of 95%.

## 3. Results and discussion

### 3.1 Descriptive analysis

A total of 157 questionnaires were completed in 5 continents with most of the respondents being female (65.6%) (Table 1). The age group of the respondents peaked at 25–34 years, which accounted for 42.7%. Data showed that most respondents came from families without children (59.9%) and 40.1% had at least 1 child under 18 years. More than half of the respondents had full-time work (52.9%) and a third were students (33.1%). In terms of educational background, 95.5% of respondents had attained college education.

**Table 1:** Demographic characteristics of respondents (n = 157).

Variable	Parameter	Frequency	Percentage
Country/Region	Australia	1	0.006
	Canada	4	0.025
	China	99	0.631
	Iran	3	0.019
	Japan	1	0.006
	Kuwait	1	0.006
	Lesotho	1	0.006
	Mongolia	3	0.019
	Netherlands	1	0.006
	New Zealand	24	0.153
	Pakistan	1	0.006
	South Africa	10	0.064
	UK	3	0.019
	USA	5	0.032
Gender	Male	54	0.344
	Female	103	0.656
Age(years)	18–24	44	0.280
	25–34	67	0.427
	35–44	23	0.146
	45–54	10	0.064
	55–64	8	0.051
	65–74	3	0.019
	75-	2	0.013
Composition of household	Children under 18 years	63	0.401
	No child	94	0.599
Employment status	Employed, working full-time	83	0.529
	Employed, working part-time	8	0.051
	Not employed, looking for work	5	0.032
	Not employed, not looking for work	1	0.006
	Student	52	0.331
	Retired	8	0.051
Highest level of education attained	Primary School or equivalent	0	0.000
	Intermediate Diploma/Certificate or Equivalent	1	0.006
	High School Diploma/Certificate or equivalent	4	0.025
	Technical skills qualification or Equivalent	2	0.013
	Bachelor’s Degree/Equivalent	63	0.401
	Master’s Degree/Equivalent	72	0.459
	Doctorates Degree (PhD)/Equivalent	15	0.096
	Medical/Health Professional	0	0.000
	Other qualifications	0	0.000

### 3.2 Construct reliability and validity

Researchers are typically interested in evaluating latent dimensions, defined as construct, that cannot be directly observed, such as anxiety, neuroticism, self-esteem, and social phobia [44]. Different from traditional statistical analysis methods, such as multiple regressions, analysis of variance and path analysis, structural equation modeling (SEM) focuses on latent variables or factors rather than observed variables. The primary purpose of SEM is to provide a mean, that isn’t affected by measurement error to estimate the structural relationship between latent variables in the theoretical model. Thus, SEM consists of two main sets of equations: measurement and structural. The measurement equations describe whether observed indicator variables are suitable as measurement means of latent variables or theoretical constructs [45–47]. Whereas the structural equations express the hypothesized relationships among the theoretical constructs that allow the assessment of the proposed theory [48].

Exploratory factor analysis (EFA) was applied to discover the factorial structure of measurement scales [49–51]. Only valid items were selected if the communalities value was >0.3 [52]. That means that a total of 5 items from food safety knowledge (K7, K8, K9, K13, and K15), 2 items from food safety attitude (A37, 38) and 2 items from food safety behavior (B17, B18) could not be included because of their communality values. Based on the selected items, the Kaisere-Meyere-Olkin (KMO) measure of sampling value for food safety knowledge, attitude and behavior were 0.662, 0.670 and 0.644 respectively, which exceeded the criterion of validity (0.60) as stated by Hair et al (2010) [52]. The reliability of all selected items was confirmed through Cronbach’s alpha coefficients that were higher than 0.5.

Confirmatory factor analysis (CFA) is usually used to verify the factor structure of latent variables. That means the selected items of the scale showed acceptable fit to the empirical data. When the observed variables are categorical, CFA is also referred to as item response theory (IRT) analysis [53, 54]. The measurement model for both CFA and SEM is a multivariate regression model that describes the relationships between a set of observed dependent variables and a set of continuous latent variables [55]. By specifying the weighted least square parameter estimation technique (WLSMV), a robust weighted least squares estimator is used. Thus, a test was carried out to further determine the construct validity of the latent factors. With respect to the convergent validity, latent factors were confirmed by evaluating factor loading (>0.30) [56]. Table 2 shows that, factor loading of food safety knowledge, attitude and behavior items ranged from 0.346 to 0.944, whilst the composite reliability value was beyond 0.70. The items with the highest factor loading provided better fit into the model for the items.

**Table 2:** Item loadings and validities for reliability test and convergent validity.

Parameter	Construct	Est. (Estimate)	SEM (Standard Error of Mean)	Est. / SEM	Two-Tailed P-value
Knowledge	K10	0.675	0.110	6.116	0.000
	K11	0.700	0.123	5.673	0.000
	K12	0.768	0.131	5.865	0.000
Attitude	A24	0.944	0.079	12.025	0.000
	A25	0.609	0.108	5.651	0.000
	A33	0.844	0.080	10.492	0.000
Behavior	B19	0.755	0.093	8.109	0.000
	B21	0.616	0.107	5.764	0.000
	B22	0.780	0.104	7.506	0.000
	B28	0.346	0.126	2.750	0.006

Only those items with higher factor loading (>0.30) were retained and introduced into the model. After the second elimination by CFA, only 3, 3, and 4 items remained in food safety knowledge, attitude and behavior, respectively. Data in Table 2 show that the latent construct of knowledge was measured by K10, K11, and K12, and the latent construct of attitude was measured by A24, A25, and A26, whilst the latent construct of behavior was measured by B19, B21, B22, and B28.

### 3.3 Measurement invariance

Measurement Invariance (MI) identifies the validity of a questionnaire, which could measure latent variables or theoretical constructs with the same structure across different groups [57]. Establishing measurement invariance involves running a set of increasingly constrained structural equation models and testing the significance of the differences between the models. The comparative indices that compare the fit of the model under consideration with fit of baseline model, for example the Tucker-Lewis Index (TLI) and Comparative Fit Index (CFI). Good fit is considered adequate if the CFI and TLI values are >0.90, and better if they are >0.95. There are absolute indices that examine closeness of fit, for example the Root Mean Square Error of Approximation (RMSEA), and SRMR (Standardized Root Mean Square Residual). The cut-off value for RMSEA is <0.08, but it is considered, better at<0.05, and it is <0.08 for the SRMR. The result of the model is shown in Table 3, while SRMR was sensitive to the size of sample and not suitable to categorical data [58].

**Table 3:** Measurement invariance.

Chi-Square	Degrees of Freedom	RMSEA	CFI	TLI	SRMR
35.206	32	0.025	0.987	0.982	0.088

### 3.4 Descriptive statistic of measurement items

Information on the nature, extent, magnitude and severity of different types of food safety problems, as well as their causes, resources and how they change over time, is essential for the development, implementation, monitoring and evaluation of effective policies and programs to improve food safety control [17]. Thus, items of food safety knowledge included the perception of COVID-19, and items of food safety attitude included interpretation of nutrition and health. Table 4 shows that the mean score of food safety knowledge was 6.64 (full score was 9), implying the food safety knowledge of the respondents on COVID-19 was rather high. Results also suggest that there was excessive worry on the health risk caused by contaminated food. The lowest score among food safety knowledge items was item K13, indicating that the majority (n = 113) of respondents were concerned about the potential transmission of the coronavirus COVID-19 through contaminated food. Compared with disinfectants, the function of ordinary soap seemed to have been ignored by many respondents. Based on the mean scores obtained for food safety attitude (full score was 14), the respondents seemed to demonstrate poor attitude on the adequacy of the amounts of fresh meat and fish (A26) consumed and regular exercises conducted (A32). Results showed that, half of the respondents changed their eating habits and tended to consume more food under lockdowns. Based on the results of data collected, over 90% of the respondents maintained their eating habits by cooking at home. All items used in accessing healthy eating behavior showed that respondents had reasonably good behavior in eating practices with all the mean value scores higher than 70% of the full score.

**Table 4:** Mean scores of items in food safety knowledge, attitude, and behavior.

Construct	Items	Mean
Food safety knowledge	K7. The coronavirus COVID-19 can be easily transmitted between among humans.	0.994
	K8. A person contaminated by coronavirus COVID-19 must undergo self-isolation, even without showing any symptoms.	0.994
	K9. The vaccination against seasonal influenza is not effective against coronavirus COVID-19.	0.892
	K10.The coronavirus COVID-19 on hands can be eliminated by hand-washing using ordinary soap.	0.529
	K11. Coronavirus on food can be killed during cooking food.	0.771
	K12. Coronavirus on surfaces can be killed by disinfectants.	0.815
	K13. The coronavirus COVID-19 cannot be easily transmitted through contaminated food.	0.280
	K14. Even when it is contaminated, food is not a health risk if it is thoroughly cooked.	0.490
	K15. Humans can catch coronavirus by touching contaminated surfaces of food packaging box.	0.879
Food safety attitude	A24.I ate a balanced diet.	0.809
	A25.I ate sufficient amounts of fresh fruit and vegetables.	0.783
	A26.I ate sufficient amounts of fresh meat and fish.	0.586
	A29. Wash hands often.	0.981
	A30. Avoid eating meat or fish not thoroughly cooked.	0.904
	A31. Avoid eating fruit or vegetable not thoroughly washed.	0.917
	A32.Exercise regularly.	0.650
	A33. Maintain a well-balanced diet.	0.828
	A34. Keep air circulating in rooms.	0.892
	A35. Avoid shaking hands with others and touching packages with bare (unprotected) hands.	0.930
	A36. Avoid going to crowded public areas.	0.975
	A37. Wear masks in public areas.	0.930
	A38. Social distancing always should be observed.	0.975
	A39. Working from home is the new normal.	0.911
Healthy eating behavior	B16. My eating habits did not change under the coronavirus covid-19 pandemic.	0.522
	B17.I maintained the same eating habits by purchasing more takeaways or fast foods.	0.866
	B18.I maintained the same eating habits by cooking more at home.	0.904
	B19.I somehow ate more food under coronavirus covid-19.	0.554
	B20.I somehow ate less food under coronavirus covid-19	0.815
	B21.I somehow ate the same amount of food under coronavirus covid-19.	0.599
	B22.I somehow ate food more frequently.	0.605
	B23.I somehow ate food less frequently.	0.828
	B27.I ate more canned foods.	0.854
	B28. I ate foods with longer shelf life.	0.611

### 3.5 Structural model

**Fig 2:**
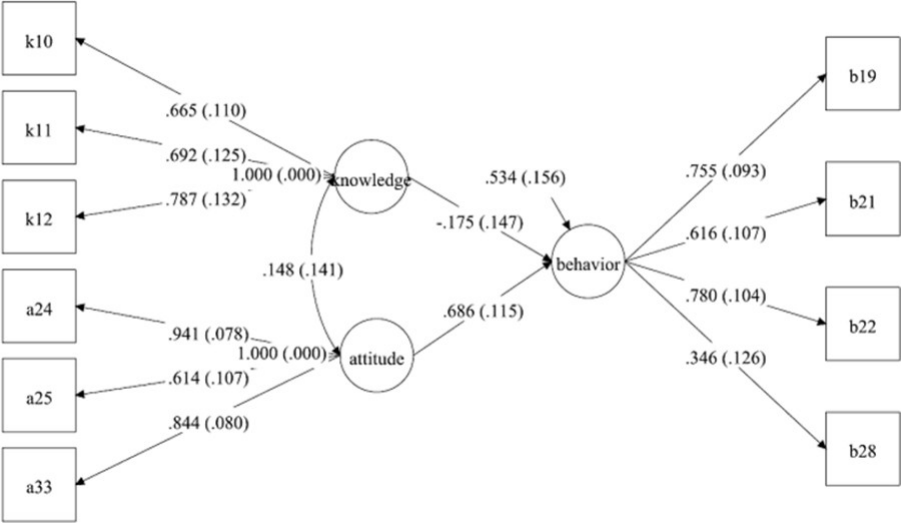
The model of food safety knowledge, attitude and eating behaviors.

The MPLUS structural model revealed the relationships among food safety knowledge, attitude and eating behavior (Fig 2). All latent variables of knowledge, attitude and behavior were confirmed by evaluating factor loading (>0.30) [47]. The results shown in Fig 2 supported H2 (the healthy eating behavior, directly affected by food safety attitude) by revealing a significant (β = 0.686, p<0.05) positive relationship between food safety attitude and healthy eating behavior. In fact, food safety attitude was the most important factor claimed by the respondents to influence their eating behavior under the COVID-19 and lockdown as shown by the high standard of β (β = 0.686) among the variables (at p< 0.05). Previous studies have confirmed that a positive attitude can lead to food safety behavior [19, 59, 60]. Hence, positive attitude under COVID-19 and lockdown could be only achieved when one believed that balanced diet and regular exercise could reduce the long-lasting adverse health outcomes, such as anxiety, frustration, panic attacks, loss or sudden increase of appetite, insomnia, depression, mood swings, delusions, fear and suicidal tendencies [61]. As illustrated in Fig 2, food safety knowledge and attitude of the respondents had very low correlations (β = 0.148). The findings obtained in the present study were similar to the results of a previous study on relationships reported by Schwardtz (1975) where knowledge and attitude independently influenced behavior. However, most studies showed that there was either a positive or negative correlation of relationship between food safety knowledge and behavior [62].

## 4. Conclusions and recommendations

The recent SARS-CoV-2 global pandemic has affected multiple aspects of our food systems, and has also provided opportunities for societies to take a close look at early topics under this crisis [63]. Overall, results of this study indicated that the respondents exhibited good attitude and good knowledge towards public health including food safety especially on the importance of social distancing, mask wearing, well-balanced diet, physical exercise and personal hygiene, such as hand washing during the pandemic lockdowns. It is revealed in this study that food safety attitude of people could positively affect healthy eating behavior. Thus, a policy to encourage broad compliance with health guidelines during a pandemic, especially among youth, could be important [64]. Of noteworthy, better food safety knowledge of the respondents did not contribute to the better eating behavior. Education can act as a long-term strategy since it appeared as a sole predictor for preventive behavioral practices toward COVID-19 in a previous survey [65]. In addition, the dissemination of information by the media can impact on consumer behavior [66]. Therefore, demographic characteristics such as age and education should be considered as moderators in further studies [67]. This study also highlighted the over-estimation of virus transmission risk to contaminated food and under-estimation of the eliminating capability of ordinary soap. The outcome of the study can provide useful insights to the authorities when considering suitable measures to prevent the impact of the COVID-19 pandemic on heal.

## 5. Limitation of this study

The findings of this study may help the policymakers to consider the attitude of the public towards food safety, inevitably some limitations may be inherent in the study and they need to be considered in interpreting the results. Firstly, since the data collected relied on self-reporting from respondents, it was assumed that the questionnaires were completed correctly. Secondly, the study was conducted among the respondents who were invited by email, Facebook, WeChat, professional websites and other social media platforms. Based on the design of the questionnaire, most of the respondents were students or staff with higher education. The researchers acknowledge that, large sample sizes are desirable and important, particularly in survey designs to improve overall quality of the data. The data were collected at the peak of the pandemic and was therefore reasonable to assume that respondents were most likely preoccupied by adopting mitigating strategies against the virus. Thus, demographic factors on relationships among food safety knowledge, attitude and eating behavior may be not easily reflected. However, the researchers also wanted to target the collection of data during this period while the experiences were still fresh in people minds. Nonetheless, we believe the sample size is reasonable and the data set can provide important insights into the interrelationships presented in this study. Further, the outcomes can form an important foundation for similar future studies in view of the persistent menace of the pandemic. Future research may include a series of studies with multiple-group modeling, and more targeted measures could be available for those specific populations.

## Supporting information

S1 Fig Title power analysis for the hypothesized model (power at 0.8). (TIF)

S2 Fig Title power analysis for the hypothesized model (power at 0.95). (TIF)

S1 File Survey questionnaire (file)/hyperlink. (PDF)

S2 File Ethics application (file). (PDF)

S3 File Title food safety knowledge, attitudes, and eating behavior under the global coronavirus pandemic. (PDF)

S1 Appendix (TXT)
