# Supplementary figures and images for: Food safety knowledge, attitudes, and eating behavior in the advent of the global coronavirus pandemic
Source: PLoS One. 2021 Dec 31;16(12):e0261832. doi: 10.1371/journal.pone.0261832 (PMC8719730; doi:10.1371/journal.pone.0261832)

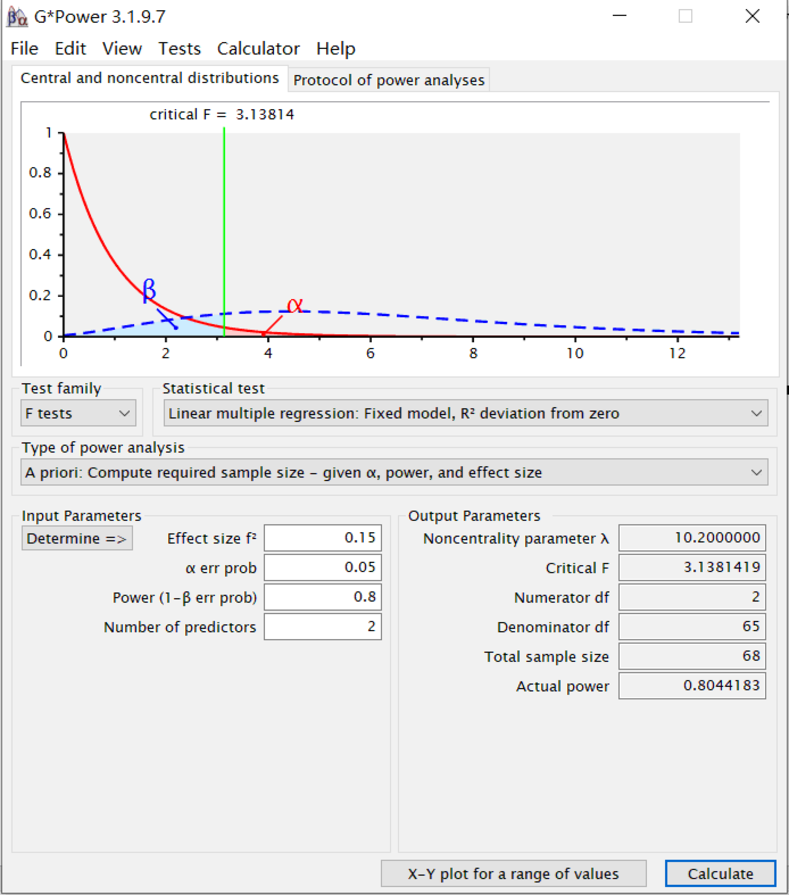

Supplement: S1 Fig — (TIF) [file pone.0261832.s001.tif]

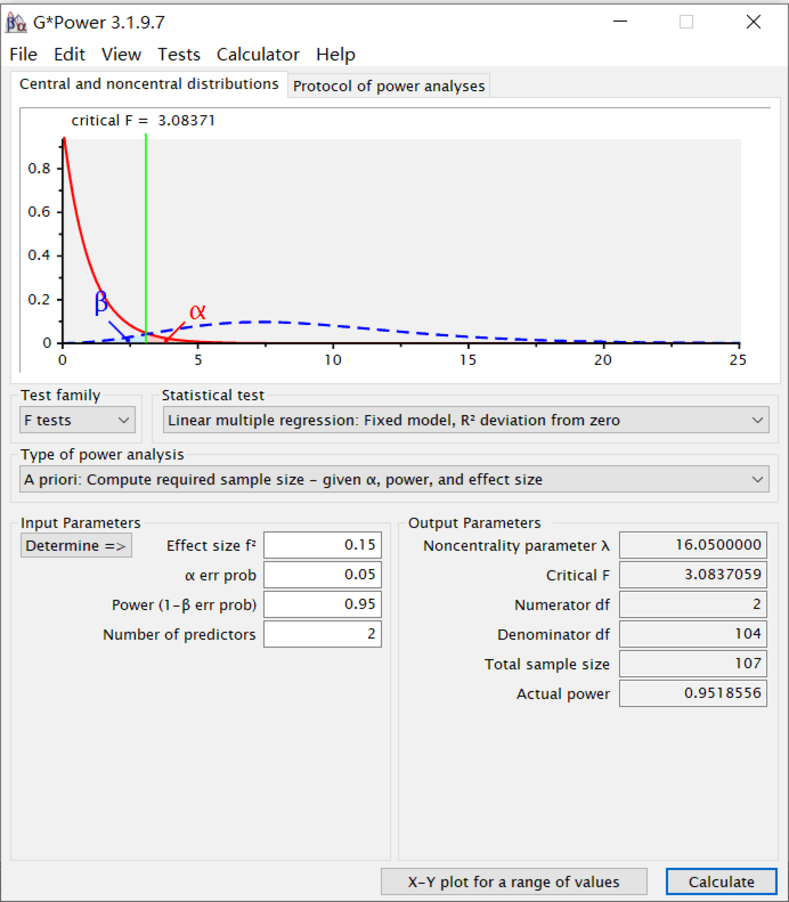

Supplement: S2 Fig — (TIF) [file pone.0261832.s002.tif]
