# Supplementary material for: Food safety knowledge, attitudes, and eating behavior in the advent of the global coronavirus pandemic
Source: PLoS One. 2021 Dec 31;16(12):e0261832. doi: 10.1371/journal.pone.0261832 (PMC8719730; doi:10.1371/journal.pone.0261832)
Supplement: S1 File — (PDF) [file pone.0261832.s003.pdf]

S1 Survey questionnaire (file)/hyperlink

Data of the research were collected through the professional online questionnaire platform of www.wjx.cn (S1 https://www.wjx.cn/jq/ 80244691.aspx).
