# Supplementary material for: Food safety knowledge, attitudes, and eating behavior in the advent of the global coronavirus pandemic
Source: PLoS One. 2021 Dec 31;16(12):e0261832. doi: 10.1371/journal.pone.0261832 (PMC8719730; doi:10.1371/journal.pone.0261832)
Supplement: S2 File — (PDF) [file pone.0261832.s004.pdf]

**From:**	humanethics@massey.ac.nz
**To:**	Mutukumira, Tony
**Cc:**	Human Ethics
**Subject:**	Human Ethics Notification - 4000022746
**Date:**	Friday, 5 June 2020 1:20:07 PM

HoU Review Group

Ethics Notification Number: 4000022746
Title: Survey on eating habits under the coronavirus (Covid-19) and lockdown in some regions of the world

Thank you for your notification which you have assessed as Low Risk.

Your project has been recorded in our system which is reported in the Annual Report of the Massey University Human Ethics Committee.

The low risk notification for this project is valid for a maximum of three years.

Please note that travel undertaken by students must be approved by the supervisor and the relevant Pro Vice-Chancellor and be in accordance with the Policy and Procedures for Course-Related Student Travel Overseas. In addition, the supervisor must advise the University's Insurance Officer.

A reminder to include the following statement on all public documents:

"This project has been evaluated by peer review and judged to be low risk. Consequently it has not been reviewed by one of the University's Human Ethics Committees. The researcher(s) named in this document are responsible for the ethical conduct of this research.
If you have any concerns about the conduct of this research that you want to raise with someone other than the researcher(s), please contact Professor Craig Johnson, Director (Research Ethics), email humanethics@massey.ac.nz. "

Please note that if a sponsoring organisation, funding authority or a journal in which you wish to publish require evidence of committee approval (with an approval number), you will have to complete the application form again answering yes to the publication question to provide more information to go before one of the University's Human Ethics Committees. You should also note that such an approval can only be provided prior to the commencement of the research.

You are reminded that staff researchers and supervisors are fully responsible for ensuring that the information in the low risk notification has met the requirements and guidelines for submission of a low risk notification.

If you wish to print an official copy of this letter, please login to the RIMS system, and under the Reporting section, View Reports you will find a link to run the LR Report.

Yours sincerely

Professor Craig Johnson
Chair, Human Ethics Chairs' Committee and
Director (Research Ethics)
