# Supplementary material for: Food safety knowledge, attitudes, and eating behavior in the advent of the global coronavirus pandemic
Source: PLoS One. 2021 Dec 31;16(12):e0261832. doi: 10.1371/journal.pone.0261832 (PMC8719730; doi:10.1371/journal.pone.0261832)
Supplement: S3 File — (PDF) [file pone.0261832.s005.pdf]

Appendix

The detailed protocol of the survey (S3 Food safety knowledge, attitudes, and eating behavior under the global coronavirus pandemic) is deposited in protocols.io with identifier (DOI) of dx.doi.org/10.17504/protocols.io.bywvpxe6.
